# Gestational Age Influences the Early Microarchitectural Changes in Response to Mechanical Ventilation in the Preterm Lamb Lung

**DOI:** 10.3389/fped.2019.00325

**Published:** 2019-08-21

**Authors:** Regina B. Oakley, David G. Tingay, Karen E. McCall, Elizabeth J. Perkins, Magdy Sourial, Peter A. Dargaville, Prue M. Pereira-Fantini

**Affiliations:** ^1^Neonatal Research Group, Murdoch Children's Research Institute, Parkville, VIC, Australia; ^2^Department of Neonatology, Royal Children's Hospital, Parkville, VIC, Australia; ^3^Department of Paediatrics, University of Melbourne, Parkville, VIC, Australia; ^4^School of Medicine and Medicinal Sciences, University College Dublin, Dublin, Ireland; ^5^Menzies Institute for Medical Research, University of Tasmania, Hobart, TAS, Australia

**Keywords:** preterm, lamb model, mechanical ventilation, lung injury, alveolarization, lung morphology

## Abstract

**Background:** Preterm birth is associated with abnormal lung architecture, and a reduction in pulmonary function related to the degree of prematurity. A thorough understanding of the impact of gestational age on lung microarchitecture requires reproducible quantitative analysis of lung structure abnormalities. The objectives of this study were (1) to use quantitative histological software (ImageJ) to map morphological patterns of injury resulting from delivery of an identical ventilation strategy to the lung at varying gestational ages and (2) to identify associations between gestational age-specific morphological alterations and key functional outcomes.

**Method:** Lung morphology was compared after 60 min of a standardized ventilation protocol (40 cm H_2_O sustained inflation and then volume-targeted positive pressure ventilation with positive end-expiratory pressure 8 cm H_2_O) in lambs at different gestations (119, 124, 128, 133, 140d) representing the spectrum of premature developmental lung states and the term lung. Age-matched controls were compared at 124 and 128d gestation. Automated and manual functions of Image J were used to measure key histological features. Correlation analysis compared morphological and functional outcomes in lambs aged ≤128 and >128d.

**Results:** In initial studies, unventilated lung was indistinguishable at 124 and 128d. Ventilated lung from lambs aged 124d gestation exhibited increased numbers of detached epithelial cells and lung tissue compared with 128d lambs. Comparing results from saccular to alveolar development (120–140d), lambs aged ≤124d exhibited increased lung tissue, average alveolar area, and increased numbers of detached epithelial cells. Alveolar septal width was increased in lambs aged ≤128d. These findings were mirrored in the measures of gas exchange, lung mechanics, and molecular markers of lung injury. Correlation analysis confirmed the gestation-specific relationships between the histological assessments and functional measures in ventilated lambs at gestation ≤128 vs. >128d.

**Conclusion:** Image J allowed rapid, quantitative assessment of alveolar morphology, and lung injury in the preterm lamb model. Gestational age-specific patterns of injury in response to delivery of an identical ventilation strategy were identified, with 128d being a transition point for associations between morphological alterations and functional outcomes. These results further support the need to develop individualized respiratory support approaches tailored to both the gestational age of the infant and their underlying injury response.

## Introduction

Continuous improvements in perinatal care have led to increased survival of premature infants (1–3). However, the neonatal morbidity associated with preterm birth remains substantial, with the lung especially susceptible to damage (3). In preterm infants the lung is structurally immature, surfactant deficient, and prone to collapse (4), necessitating the application of respiratory support to ensure survival. However, positive pressure ventilation and supplemental oxygen can disrupt the intricate molecular networks responsible for normal distal lung development (5, 6). The resultant alveolar simplification reduces gas exchange resulting in increased oxygen need (7).

The developmental state of the lung at birth strongly influences clinical outcomes. In both infant (8) and lamb studies (9, 10) increasing prematurity is associated with reduced oxygenation, impaired lung compliance, and greater lung injury. At the protein level, we have recently identified clear distinctions between the plasma proteome response of early preterm, preterm and term lambs exposed to an identical ventilation strategy (10). However, characterization of gestational-age specific alterations in lung histology across a range of prematurity outcomes has not been performed. Rather, previous studies have assessed ventilation-associated morphological alterations within a narrowly defined developmental stage (11, 12), or in the context of a clinical diagnosis such as BPD or Hyaline Membrane Disease (13–16). Understanding the influence of gestational age at ventilation on lung injury pathogenesis, including alterations in lung morphology, is essential to the development of individualized treatment strategies aimed at protecting the preterm lung.

The ventilated preterm lamb model is an established pre-clinical model for the study of both respiratory therapies and subsequent lung injury, sharing key morphological, mechanical, and developmental similarities with preterm infants. It has proven to be a useful physiological model as it mirrors the clinical setting of preterm birth and respiratory failure that requires ventilation support with oxygen-rich gas (17). The preterm lamb model has been extensively employed to study the pathological impact of mechanical ventilation at a single developmental stage using “score” based injury assessment tools (18, 19). By reducing the assessment to either presence or absence of injury (18) or a 0–4 point scale (19), these approaches prevent detection of subtle, developmental-specific pathological alterations. Furthermore, they are highly susceptible to inter and intra-operator error which limits validity and comparison between studies.

Advances in the quantitative histological assessment of lung structure have the capacity to address these limitations and thereby significantly enhance our understanding of the influence of gestational age at ventilation on preterm lung architecture. Notable amongst these has been the development of the open-access software “ImageJ” which facilitates the development of automated, quantitative, stereological based methods. Our primary objective was to quantify the impact of gestational age on lung injury development. To achieve this objective we developed a semi-automated method of histological analysis to assess the changes in alveolar morphology associated with lung injury in preterm lambs of gestational age 119–140 days that were exposed to an identical ventilation strategy. Our secondary objective was to identify associations between gestational age-specific morphological alterations and key functional outcomes.

## Materials and Methods

All experiments were performed at the large animal facility of the Murdoch Children's Research Institute (Melbourne, Australia). They were conducted according to the guidelines of the National Health and Medical Research Council (Australia), and with prior approval from the Animal Ethics Committee of the Murdoch Children's Research Institute (Melbourne, Australia). These studies were performed as part of a larger program examining the role of ventilation strategy at birth on lung mechanics, gas exchange and lung injury in the preterm lung (9, 20, 21). The antenatal management and ventilation strategy, and justifications, have been reported in detail before (7, 9, 20–24).

### Study 1: Examination of the Impact of Ventilation on Lambs Within the Early Saccular Stage: Morphological Outcomes at 124d and 128d Gestation

As increasing prematurity has been associated with increasing evidence of lung injury (8) we first focused our histological studies on the gestational age groups most likely to exhibit significant morphological alteration, those within the early saccular stage, to establish the utility of the methods for differentiation of histological outcomes.

### Antenatal Management

Date-mated Border-Leicester/Suffolk lambs, mated from the same flock over one winter, were delivered via cesarean section under general anesthesia at 124 ± 1d (*n* = 17) or 128 ± 1d (*n* = 21). Consistent with clinical practice (25) all preterm groups were exposed to maternal 11.4 mg IM betamethasone 24 at 48 h before delivery. At birth lung fluid was passively drained and birth weight recorded. Lambs were randomly assigned to receive no ventilation (*n* = 5/group; unventilated control group) or an initial sustained inflation delivered at 40 cm H_2_O until full lung aeration was visualized using real-time electrical impedance monitoring (9, 20) and then positive pressure ventilation (PPV; ventilated group) (SLE5000, SLE UK Ltd, South Croyden UK) in a volume-targeted ventilation mode as described previously (7, 9, 20–22). FiO_2_, tidal volume (5.5–8 ml/kg) and ventilator rate (40–60 inflations/minute) were adjusted as appropriate using a standardized protocol to maintain SpO_2_ between 88 and 95% and partial arterial pressure of carbon dioxide between 45 and 60 mmHg (7, 9, 20, 21). At 60 min, lambs were ventilated with a FiO_2_ of 1.0 for 3 min and disconnected to atmosphere for 2 min. At the end of the study period lambs received a lethal IV dose of pentobarbitone immediately prior to defining the pressure-volume (PV) curve of the lung using the super syringe method and the whole lung then removed.

### Lung Histology

Following removal of the whole lung, the right upper lobe was inflation fixed with 4% paraformaldehyde (Australian Biostain Pty Ltd, Traralgon, Australia) for 90 min at 20 cmH_2_O, followed by submersion in 4% paraformaldehyde for 24 h. Samples were obtained from three standardized sites per lung and placed in Zamboni's fixative for 24 h prior to processing and embedding in paraffin.

**Figure 1:**
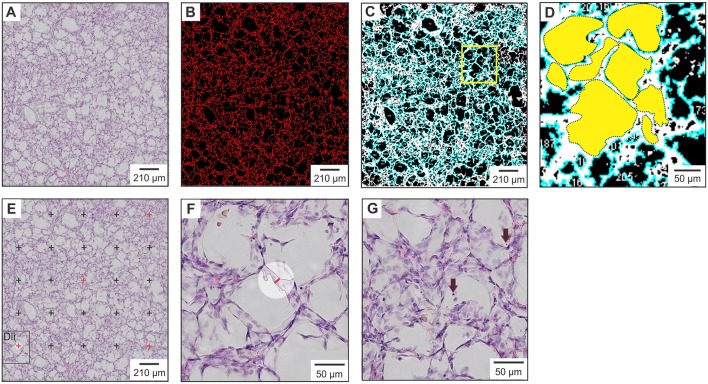
Histological measures of lung injury. **(A)** Raw image cropped to size prior to processing and analysis with Image J. **(B)** Assessment of % lung tissue with measured areas of lung tissue highlighted in red. **(C)** Alveolar surface area measurements with alveolar units highlighted in blue. **(D)** Magnification of yellow square in panel **(C)**. Dotted outlines outline each alveolar unit, the area of which is shown in solid yellow. **(E)** Septal width measured at the five cross-points denoted in red. **(F)** Magnification of the black square in panel **(E)**, showing a septal width measurement (red line) made at the previously indicated cross-point. **(G)** Magnified image in which detached epithelial cells located within the alveoli are indicated by black arrows.

Histological examination of the right upper lobe sample was performed on 4 μm formalin-fixed lung sections stained with hematoxylin and eosin. Post-staining, whole section images were obtained using a slide scanner (Olympus, Tokyo, Japan), cropped to image size 1.50 × 1.37 mm (L × W; Figure 1A) using Adobe Photoshop software (Adobe Systems Inc., San Jose, USA) and analyzed using Image J software (US National Institute of health, https://imagej.nih.gov/nih-image/) (26). Following setting of the image scale, five putative morphological measures of lung injury (% lung tissue, number of alveoli/image, total alveolar area/image, average alveolar area/image, and variation in alveolar area) were quantitatively assessed using an automated method (see Supplementary Material for the ImageJ macro code plugin). In brief, assessment of % lung tissue was performed using the “threshold macro” function (Figure 1B). An automated macro code plugin based on the “analyze particles” function (Supplementary Material) was used to determine number of alveoli/image, total alveolar surface area/image, and average alveolar area/image (Figures 1C,D). The coefficient of variation (CoV) in alveolar area (standard deviation/mean) was determined to report the variation in alveolar area. Two additional measures, alveolar septa width and number of detached epithelial cells, could not be accurately quantitated with automation and subsequently were manually assessed in Image J following application of a 25-point grid to the cropped image. Septal width was measured at five standardized cross-points using the line selection tool (Figures 1E,F). The total number of detached epithelial cells was counted using the point selection tool (Figure 1G). For each animal, the mean value obtained from assessment of three standardized lung region samples is reported. Manual measures are reported as the geometric mean of results obtained from two blinded observers, with <10% difference in result observed between observers.

### Study 2: Comparison of Ventilated Lung Morphology From Early to Late Saccular Developmental: Morphological Outcomes Between 119 and 140d

In Study 2 additional gestational age groups were studied, including animals prior to 124d and beyond 128d gestations, thereby allowing mapping of ventilated lung morphology from the earliest saccular stage through to an early alveolar stage. Lambs of gestational ages 119 ± 1d (*n* = 10), 124 ±1d (*n* = 12), 128 ±1d (*n* = 16), 133 ±1d (*n* = 10), and 139–140d (*n* = 11) were delivered, managed and then underwent histological assessment as per the Study 1 ventilation groups, however as this study utilized data from a larger study with a different primary outcome, age-matched unventilated controls were unavailable for all gestational age cohorts.

### Study 3: Examination of the Relationship Between Ventilation-Induced Morphological Alterations and Functional Assessments of Lung Function and Injury in Term and Near-Term Lambs (Gestational Ages 140 and 133d) and in Preterm Lambs (119, 124, and 128d)

Results obtained during Study 2 were re-compiled into two developmental sub-groups: term, near-term (gestational ages 140 and 133d) and preterm (119, 124, and 128d), representing the fetal alveolarized lung and saccular lung, respectively. Data regarding birth-related factors, and measurements obtained at 60 min post-ventilation including ventilator parameters, measures of gas exchange, measures of lung mechanics, and biochemical and molecular markers of lung injury were gathered as previously detailed (10).

### Statistical Analysis

These analyses were performed as part of a series of studies investigating the effects of sustained lung inflation strategies on physiological and injurious outcomes at different gestations, and sample numbers were derived from power calculations in relation to the specific aims of those studies (9, 10, 21). No lambs were excluded from analysis in the studies reported herein.

All statistical and modeling analysis was performed with GraphPad PRISM 7 (GraphPad Software, SanDiego, CA) and *P* < 0.05 considered significant. After testing for normality, data were reported as mean ± standard deviation (SD) or median (interquartile range, IQR), and confidence levels and 95% confidence intervals of the mean determined. Significant differences in histological indices between ventilated and non-ventilated groups (Study 1) and gestation groups (Study 2) were sought using parametric and non-parametric ANOVA as appropriate (one-way ANOVA or Kruskall-Wallis ANOVA, respectively) with *post hoc* testing to identify intergroup differences as necessary (Tukey's *post hoc* test or Dunn's multiple comparison test, respectively). To test how histological indices co-varied with other measures including birth-related factors, functional assessments, and molecular markers of lung injury within each developmental stage (Study 3), Pearson (parametric data), or Spearman's (non-parametric data) correlation coefficients were calculated and heatmaps indicative of the strength of association were generated.

## Results

### Examination of the Impact of Ventilation on Lambs Within the Early Saccular Stage: Morphological Outcomes at 124 and 128d Gestation

No ewes had evidence of sepsis or chorioamnionitis. Ventilated lambs at 124d weighed less at birth than gestation-matched unventilated controls (*P* = 0.02; Table 1). Gender was comparable between all study groups however plurality was discordant between 124d ventilated (all twins) and unventilated control groups (all triplets).

**Table 1:** Birth characteristics of the Study 1 comparison groups.

	**124d group**		**128d group**	
	**Unventilated**	**Ventilated**	**Unventilated**	**Ventilated**
No. lambs studied	5	12	5	16
Gestational age (days)	125 (124, 125)	124 (123,125)	127 (127,127)	128 (127,129)
Birth weight (kg)	3.6 (3.3, 4.9)	3.1 (2.4, 3.6)^*^	3.1 (2.6, 3.5)	3.3 (2.2, 4.3)
Gender	3F: 2M	6F: 6M	2F: 3M	9F: 7M
Plurality	Five triplets	Twelve twins	One single: three twins: one triplet	one singleton: 15 twins

*Median (range)*.

* *Differs from the (corresponding) unventilated group, P < 0.05, ANOVA with Tukey's post hoc test*.

**Figure 2:**
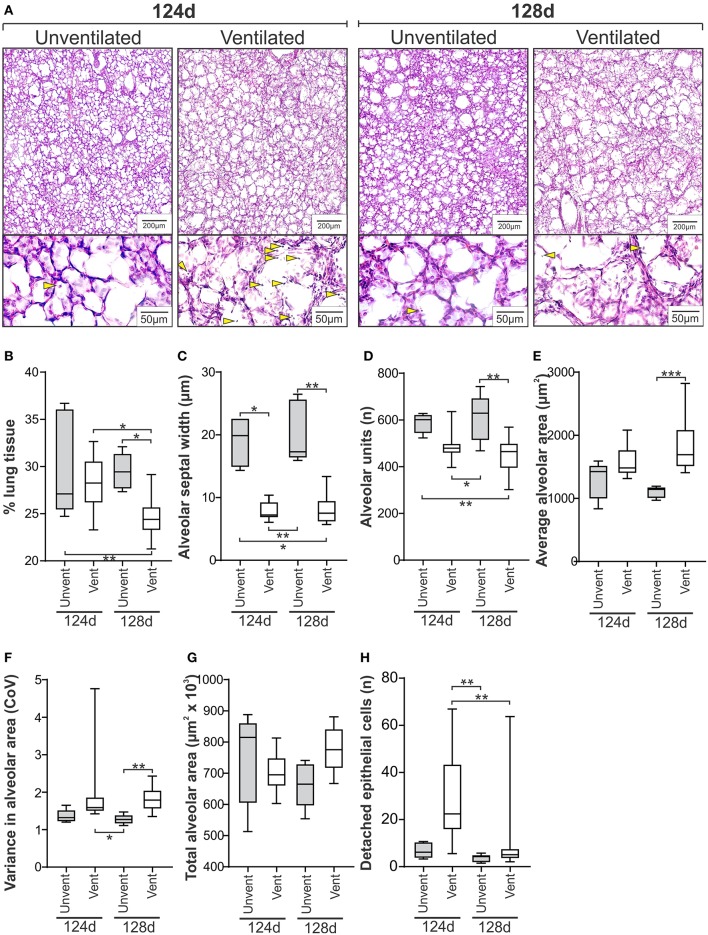
Histological measures of lung injury in the ventilated and unventilated lung (*Study 1*). **(A)** Representative H&E stained micrographs of lung tissue from unventilated and ventilated lambs at 124d and 128d gestation at low and high magnification exemplifying lung morphology and presence of detached epithelial cells (lower panel, blunted yellow arrows). Quantitative histological assessments shown for % lung tissue **(B)**, alveolar septal width **(C)**, number of alveoli per image **(D)**, average alveolar area **(E)**, variance in alveolar area **(F)**, total alveolar area per image **(G)**, and number of detached epithelial cells per image **(H)**. The boxes encompass the 25th to 75th percentiles and line within the box the median. Whisker caps denote the maximum and minimum values. ^*^P < 0.05, ^**^P < 0.01, ^***^P < 0.001 as determined by parametric or non-parametric ANOVA as appropriate [one-way ANOVA; **(B,D,G)** or Kruskall-Wallis ANOVA; **(C,E,F,H)**] with *post hoc* testing to identify intergroup differences (Tukey's *post hoc* test or Dunn's multiple comparison test, respectively).

Representative micrographs from Study 1 are shown in Figure 2A. The confidence level varied between 93.8 and 98.7% across the study groups, with confidence intervals for each measure reported in Supplementary Table 1. Unventilated lung from lambs aged 124 and 128d was indistinguishable morphologically for the seven assessment measures (Figures 2B–H). Differences between unventilated and ventilated lung were more prominent at 128d with decreased lung tissue (*P* = 0.01), alveolar septal width (*P* = 0.0042), and number of alveoli/image (*P* = 0.0023) observed together with increased average alveolar area (*P* = 0.0009) and variance in alveolar area (*P* = 0.0066) in ventilated lung when compared with unventilated lung. At 124 ± 1d, only alveolar septal width was significantly increased in ventilated lung when compared with gestation-matched unventilated lung (*P* = 0.01). Differences observed between the two ventilated groups included increased lung tissue (*P* = 0.01) and more detached epithelial cells (*P* = 0.0070) in the less mature animals at 124d compared with those at 128d.

### Study 2: Comparison of Ventilated Lung Morphology From Early Saccular to Early Alveolar Stage: Morphological Outcomes Between 119 and 140d

To gain greater insight into the impact of developmental stage on the morphological response to ventilation, we expanded our assessment of ventilated-associated morphological alterations to include lung representing the early saccular development through to early alveolar development (119–140d gestation). No ewes included in Study 2 had evidence of sepsis or chorioamnionitis. Birth weight increased with gestational age (Table 2). Lambs included in gestational age groups 119–128d were comparable for gender, however the 133d and 140d group included a higher proportion of males. Plurality and birth order were comparable for lambs in all GA study groups excluding the 140d group which also included triplet births.

**Table 2:** Birth characteristics of the Study 2 comparison groups.

	**119d**	**124d**	**128d**	**133d**	**140d**
No. lambs studied	10	12	16	10	11
Gestational age (days)	119 (118, 120)	124 (123, 125)	128 (127, 129)	133 (132, 134)	139 (139, 140)
Birth weight (kg)	2.57 ± 0.39	3.05 ± 0.34	3.28 ± 0.50	4.03 ± 0.68	4.66 ± 0.56
Gender	4F: 6M	6F: 6M	9F: 7M	3F: 7M	3F: 8M
Plurality	Two singletons: eight twins	Twelve twins	One singleton: 15 twins	Ten twins	One singleton: four twins: six triplets

*Mean ± SD or median (range) as appropriate*.

**Figure 3:**
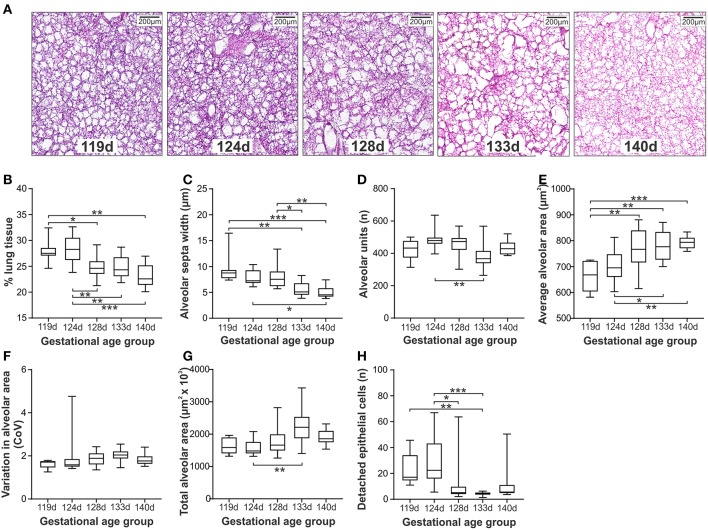
Histological measures of lung injury in ventilated lambs at different gestations (*Study 2*). Representative micrographs at gestations 119d to 140d **(A)**. Morphological assessments included; % lung tissue **(B)**, alveolar septa width **(C)**, number of alveoli per image **(D)**, average alveolar area **(E)**, variation in the alveolar area **(F)**, total alveolar area in each image **(G)**, and the number of detached epithelial cells per image **(H)**. The boxes encompass the 25th to 75th percentiles and line within the box the median. Whisker caps denote the maximum and minimum values. ^*^P < 0.05, ^**^P < 0.01, ^***^P < 0.001 as determined by parametric or non-parametric ANOVA as appropriate [one-way ANOVA; **(B,E)** or Kruskall-Wallis ANOVA; **(C,D,F–H)** with *post hoc* testing to identify intergroup differences (Tukey's *post hoc* test or Dunn's multiple comparison test, respectively).

Representative micrographs of ventilated lung tissue at gestations from 119d to 140d are shown in Figure 3A. The confidence level across the gestational ages varied from 96.1 to 98.4% (Supplementary Table 2). A clear differentiation was observed between the lambs at gestations ≤124d compared with those at 128d and above (Figures 3B–H), with the less mature GA groups exhibiting increased lung tissue (Figure 3B), and decreased average alveolar area (Figure 3E). Alveolar septa width was, in general, increased in lambs between 119 and 128d when compared with lambs at or beyond 133d (Figure 3C). The number of alveoli per scanned image was decreased (Figure 3D) and conversely the total alveolar area per scanned image increased in 124d lambs compared with the 133d group (Figure 3G). No difference in variation in alveolar area was observed amongst the gestational age groups (Figure 3F). The number of detached epithelial cells exhibited a similar pattern to other measures, with increased numbers of epithelial cells observed in the 119d group (relative to 133d) and in the 124d group (relative to 128 and 133d; Figure 3H).

### Study 3: Examination of the Relationship Between Histological Lung Injury Markers and Other Measures of Lung Injury

**Figure 4:**
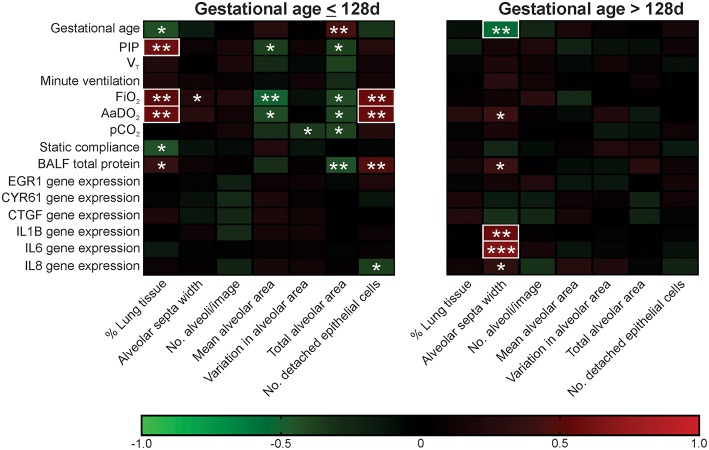
Association between histological assessments and functional measures in ventilated lambs of gestational age ≤128 d and >128 d. Red squares represent positive associations and green squares negative associations with color intensity indicative of strength of association (as shown in the color scale). *R* values of <-0.5 or >0.5 are highlighted with a white box. Parametric data sets were analyzed using Pearson correlation coefficient and non-parametric data sets analyzed using Spearman's correlation coefficient; ^*^P < 0.05, ^**^P < 0.01, ^***^P < 0.001.

Two clear patterns emerged in the histological assessments in Study 2, with a transition zone around 128d gestation. These findings were mirrored in the measures of gas exchange, lung mechanics, and molecular markers of lung injury (Supplementary Table 3). In Study 3 correlation analysis was used to examine the gestation-specific relationships between the seven histological assessments and measures of function and molecular indicators of lung injury in ventilated lambs at gestation ≤128 and >128d (Figure 4).

A larger number of significant associations were detected in ventilated lambs aged ≤128d when compared with those aged >128d (21 vs. 6). Furthermore, whilst 6/7 histological assessments were correlated with lung function and/or molecular injury markers in lambs at gestations ≤128d, only alveolar septa width was linked to these measures beyond 128d. In ventilated lambs ≤128d, % lung tissue, total alveolar area, and number of detached epithelial cells were associated with the alveolar-arterial oxygen difference (AaDO_2_) and bronchoalveolar lavage fluid (BALF) total protein concentration. In contrast, in ventilated lambs >128d, alveolar septa width was positively correlated with relative gene expression of IL1B, IL6, and IL8, known markers of lung injury and inflammation.

## Discussion

Although necessary to sustain life following preterm birth, mechanical respiratory support and supplemental oxygen can disrupt the intricate molecular networks responsible for normal distal lung development and initiate lung injury. This results in significantly reduced pulmonary function which continues well beyond the initial neonatal period (27–31). Histological studies of post-mortem tissue from ventilated preterm infants consistently describe a pulmonary morphology characterized by enlarged alveoli with blunted secondary septa, increased septal thickness, blunted pulmonary microvasculature growth, and thickened alveolar-capillary membranes (32–34). Functionally, these alterations present a substantial anatomic barrier to diffusion of oxygen and carbon dioxide (17), thereby limiting provision of adequate gas exchange. The preterm lamb is a widely accepted model for the study of ventilation associated changes in pulmonary morphology and is commonly used in the pre-clinical development of therapies aimed at minimizing damage whilst optimizing gas exchange (7, 35, 36). However, few studies have included quantitative morphological assessment of the lung, or considered the impact of gestational age on microarchitectural alterations, limiting comparison across studies. The availability of automated analysis using software like ImageJ provides the opportunity for greater consistency and reliability in measuring lung injury. To our knowledge this is the first study to report the use of a semi-automated software toolkit to evaluate changes in lung histology in experiments conducted across the spectrum of gestational ages.

In Study 1, we confirmed that the suite of seven histological measures could differentiate between unventilated and ventilated lung tissue in lambs at either 124d (early saccular phase; 24 weeks human equivalency) or 128d (mid saccular phase; 29 weeks human equivalency). Unventilated controls at 124 and 128d were morphologically indistinguishable, however clear differences were observed between 124 and 128d ventilated lamb groups including a 5-fold increase in the number of detached epithelial cells and a statistically significant but minor decrease in % lung tissue in 124d ventilated lambs compared with 128d ventilated lambs. When comparing between gestation-matched ventilated and unventilated lung tissue, differences in morphology were more often observed in 128d lambs when compared to 124d lambs. Ventilation-induced alterations at 124d included a 2.8-fold reduction in alveolar septa width and a 3.7-fold increase in the numbers of detached epithelial cells reflective of barotruma (excessive pressure on alveolar units). Conversely, alterations at 128d, including increased variation in alveolar size and average alveolar area combined with decreased % lung tissue, alveolar septal width, and number of alveoli, may reflect a more complex mechanotrauma response which includes both atelectotrauma (uneven ventilation of alveolar units) and volutrauma (over inflation of alveolar units).

Gestational age at time of ventilation has been shown to influence the trajectory of aeration at birth (9), and the plasma proteome response (10). Extremely preterm infants in the transitionary period from the canalicular to saccular phases of lung development are more susceptible to stretch-related injury (37) and disrupted alveolar development (38). In Study 2, histological assessment of lung from lambs ventilated at very early (119d), early- (124d), mid- (128d), late-mid (133d), or late-saccular (140d) developmental stages confirmed gestation-specific alterations in alveolar structure and lung injury detection following 60 min of standardized mechanical ventilation from birth. In lambs ventilated within the early saccular period (124d) the number of detached epithelial cells and % lung tissue was increased, whilst mean alveolar area was decreased when compared with lambs within the mid- to late-saccular period (>128d). Thicker alveolar septum was observed in lambs at lower gestations which may be indicative of injury-related increases in collagen deposition and inflammatory cell influx as observed in other pulmonary diseases including emphysema (39). This greater propensity for alveolar damage within the early saccular phase was reflected in physiological and molecular indices, with AaDO_2_ (a measure of the integrity of the alveolar capillary unit) on average 4.5-fold higher in lambs of gestational age ≤124d vs. either 133 or 140d groups, and the relative gene expression of IL6 on average 3.8-fold higher in lambs ≤128d compared with lambs 133 or 140d lambs. Previous studies have confirmed that whilst surfactant composition is comparable amongst gestational age groups aged 124–145d, lung maturation is associated with an increase in endogenous surfactant production (40). Furthermore, the efficacy of delivered exogenous surfactant has recently been shown to be influenced by administration of mechanical ventilation (21). Hence our results of increasing injury with prematurity likely reflect a reduced capacity for surfactant-led protection within the immature lung.

A strength of the current study was the correlation of morphological alterations with gas exchange, lung mechanics, and molecular markers of injury during early-mid saccular development (119, 124, and 128d groups) and early alveolar development (128, 133, and 140d groups). The patterns of association were unique to the two developmental stages, with the links between histological markers and other outcomes being strongest in the more immature animals. In lambs ventilated in the early-mid saccular stages, histological assessments of alveolar structure (e.g., total and mean alveolar area) were negatively correlated with ventilator parameters (PIP), gas exchange (AaDO_2_) and lung fluid protein concentration (a general injury measure), conversely the number of detached epithelial cells detected and the % lung tissue were positively correlated against the same functional measures. This may reflect a clinical scenario in which greater inflation pressure and supplementary oxygen is required in lambs with reduced alveolar surface area but results in increased damage of the alveolar diffusion barrier (i.e., increased numbers of detached epithelial cells). In contrast, in lambs ventilated in the mid-late saccular stage of lung development, the histological feature linked to other outcomes was increased alveolar septa width, which was associated with worse oxygenation and increased inflammatory markers. It may be that ventilation beyond 128 days initiates an inflammatory-based injury response, leading to thicker septal widths that limit the efficiency of the air-blood barrier. Importantly, the unique association patterns identified in lambs of gestational age ≤128d vs. lambs beyond 128d emphasize a need to develop histological assessment tools suited to the specific pathological outcome of that developmental stage.

### Limitations

As this study utilized data from a larger study with a different primary outcome, age-matched unventilated controls were unavailable for all gestational age cohorts. Whilst this clouds the interpretation of expected age-specific alveolar alterations such as decreased lung tissue, other recorded alterations such as increasing numbers of detached epithelial cells with increasing prematurity are suggestive of developmental-specific response patterns. Furthermore, the two unventilated groups utilized in Study 1 contained relatively small sample sizes and were impacted by a level of variation, albeit sample sizes were still larger than the majority of preterm lamb studies (41–51). There was also a high twin and triplet rate as reflected by the higher birth weight observed in unventilated 124d lambs compared with their gestation-match ventilated counterparts. We did not examine indicative fluid absorption parameters such as epithelial sodium channel status, and therefore cannot delineate the impact of lung fluid reabsorption during the birth transition on gestational-age specific injury outcomes. Consistent with most other studies of lung injury in the preterm lamb mechanical ventilation was brief, being limited to 60 min (9, 10, 22, 52–55). This may preclude extrapolation of our histological findings to long term models, but does establish a methodological approach that can be applied across all models. Whilst this study characterized a number of alveolar-related parameters commonly observed in the “new” BPD scenario such as changes in septal width and alveolar size (56), we did not include morphological assessment of alternative cellular alterations such as changes in vascular remodeling or neuronal networks. Future studies focusing on detailing the range of cellular responses will be important for providing a complete picture detailing the impact of ventilation on preterm lung morphology.

## Conclusion

We report a method for the comprehensive histological assessment of lung morphology following ventilation in the preterm lamb. Importantly, this method allows for the quantitative, rapid assessment of alveolar morphology, and lung injury in the preterm lamb model, potentially avoiding inter- and intra-operator error, and allowing for reliable comparisons between studies. Importantly these measures identified nuances in the morphological response to ventilation of the lung at early-mid and mid saccular-early alveolar developmental stages, making them highly useful to the future development of treatment strategies specific for the developmental stage of the lung and essential for the accurate interpretation of pre-clinical studies performed using the preterm lamb model. The observed gestational age-specific patterns of injury in response to delivery of an identical ventilation strategy further support the need for the development of individualized respiratory support approaches which are tailored to both the gestational age of the infant and their underlying pathology.

## Data Availability

All datasets generated for this study are included in the manuscript and/or the Supplementary Files. Raw data is available upon request from the corresponding author.

## Ethics Statement

All experiments were performed at the large animal facility of the Murdoch Children's Research Institute (Melbourne, Australia). They were conducted according to the guidelines of the National Health and Medical Research Council (Australia), and with prior approval from the Animal Ethics Committee of the Murdoch Children's Research Institute (Melbourne, Australia).

## Author Contributions

RO, PP-F, and DT contributed to study conception and design. RO was responsible for method development, data acquisition, and statistical analysis. RO and PP-F were equally responsible for writing of the main manuscript text. PP-F supervised the study, assisted with interpretation of the data, and critical revision for important intellectual content. DT and PD provided critical revision for important intellectual content, obtained funding, and supervised the study. PP-F, DT, KM, EP, and MS were involved in all lamb experimental work. All authors contributed to critical revision of the manuscript.

### Conflict of Interest Statement

The authors declare that the research was conducted in the absence of any commercial or financial relationships that could be construed as a potential conflict of interest.

## References

[1] Field DJ Dorling JS Manktelow BN Draper ES. Survival of extremely premature babies in a geographically defined population: prospective cohort study of 1994-9 compared with 2000-5. BMJ. (2008) 336:1221–3. 10.1136/bmj.39555.670718.BE 18469017 PMC2405852

[2] Goldenberg RL Jobe AH. Prospects for research in reproductive health and birth outcomes. JAMA. (2001) 285:633–9. 10.1001/jama.285.5.633 11176872

[3] Saigal S Doyle LW. An overview of mortality and sequelae of preterm birth from infancy to adulthood. Lancet. (2008) 371:261–9. 10.1016/S0140-6736(08)60136-1 18207020

[4] Subramaniam P Ho JJ Davis PG Prophylactic nasal continuous positive airway pressure for preventing morbidity and mortality in very preterm infants. Cochrane Database Syst Rev. (2016) 14:CD001243 10.1002/14651858.CD001243.pub3 27315509

[5] Bjorklund LJ Ingimarsson J Curstedt T John J Robertson B Werner O. Manual ventilation with a few large breaths at birth compromises the therapeutic effect of subsequent surfactant replacement in immature lambs. Pediatr Res. (1997) 42:348–55. 10.1203/00006450-199709000-00016 9284276

[6] Dargaville PA Tingay DG. Lung protective ventilation in extremely preterm infants. J Paediatr Child Health. (2012) 48:740–6. 10.1111/j.1440-1754.2012.02532.x 22970667

[7] Tingay DG Rajapaksa A Zonneveld CE Black D Perkins EJ Adler A. Spatiotemporal aeration and lung injury patterns are influenced by the first inflation strategy at birth. Am J Respir Cell Mol Biol. (2016) 54:263–72. 10.1165/rcmb.2015-0127OC 26186685

[8] Jobe H. Mechanisms of lung injury and bronchopulmonary dysplasia. Am J Perinatol. (2016) 33:1076–8. 10.1055/s-0036-1586107 27603539

[9] McCall KE Waldmann AD Pereira-Fantini P Oakley R Miedema M Perkins EJ. Time to lung aeration during a sustained inflation at birth is influenced by gestation in lambs. Pediatr Res. (2017) 82:712–20. 10.1038/pr.2017.141 28604757

[10] Pereira-Fantini PM Byars SG McCall KE Perkins EJ Oakley RB Dellaca RL. Plasma proteomics reveals gestational age-specific responses to mechanical ventilation and identifies the mechanistic pathways that initiate preterm lung injury. Sci Rep. (2018) 8:12616. 10.1038/s41598-018-30868-x 30135517 PMC6105628

[11] Albertine KH Jones GP Starcher BC Bohnsack JF Davis PL Cho SC. Chronic lung injury in preterm lambs. disordered respiratory tract development. Am J Respir Crit Care Med. (1999) 159:945–58. 10.1164/ajrccm.159.3.9804027 10051278

[12] Coalson JJ Kuehl TJ Escobedo MB Hilliard JL Smith F Meredith K. A baboon model of bronchopulmonary dysplasia. II. Pathologic features. Exp Mol Pathol. (1982) 37:335–50. 6924896 10.1016/0014-4800(82)90046-6

[13] Hislop A Haworth SG. Pulmonary vascular damage and the development of cor pulmonale following hyaline membrane disease. Pediatr Pulmonol. (1990) 9:152–61. 2148977 10.1002/ppul.1950090306

[14] Husain N Siddiqui NH Stocker JT. Pathology of arrested acinar development in postsurfactant bronchopulmonary dysplasia. Hum Pathol. (1998) 29:710–7. 9670828 10.1016/s0046-8177(98)90280-5

[15] Margraf LR Tomashefski JF Jr Bruce MC Dahms BB. Morphometric analysis of the lung in bronchopulmonary dysplasia. Am Rev Respir Dis. (1991) 143:391–400. 10.1164/ajrccm/143.2.391 1990959

[16] Hislop A Wigglesworth JS Desai R Aber V. The effects of preterm delivery and mechanical ventilation on human lung growth. Early Hum Dev. (1987) 15:147–64. 3608888 10.1016/0378-3782(87)90003-x

[17] Albertine KH. Progress in understanding the pathogenesis of BPD using the baboon and sheep models. Semin Perinatol. (2013) 37:60–8. 10.1053/j.semperi.2013.01.001 23582959 PMC3664547

[18] Corsini DI Burchielli S Cangiamila V Longini M Paternostro F Buonocore G. Natural surfactant combined with beclomethasone decreases oxidative lung injury in the preterm lamb. Pediatr Pulmonol. (2009) 44:1159–67. 10.1002/ppul.21145 19911365

[19] Church JT Coughlin MA Perkins EM Hoffman HR Barks JD Rabah R. The artificial placenta: continued lung development during extracorporeal support in a preterm lamb model. J Pediatr Surg. (2018) 53:1896–903. 10.1016/j.jpedsurg.2018.06.001 29960740 PMC6151273

[20] Tingay G Lavizzari A Zonneveld CE Rajapaksa A Zannin E Perkins E. An individualized approach to sustained inflation duration at birth improves outcomes in newborn preterm lambs. Am J Physiol Lung Cell Mol Physiol. (2015) 309:L1138–49. 10.1152/ajplung.00277.2015 26408555

[21] Tingay G Pereira-Fantini PM Oakley R McCall KE Perkins EJ Miedema M. Gradual aeration at birth is more lung protective than a sustained inflation in preterm lambs. Am J Respir Crit Care Med. (2019). 10.1164/rccm.201807-1397OC. [Epub ahead of print]. 30730759

[22] Tingay G Bhatia R Schmolzer GM Wallace MJ Zahra VA Davis PG. Effect of sustained inflation vs. stepwise PEEP strategy at birth on gas exchange and lung mechanics in preterm lambs. Pediatr Res. (2014) 75:288–94. 10.1038/pr.2013.218 24257321

[23] Tingay DG Wallace MJ Bhatia R Schmolzer GM Zahra VA Dolan MJ. Surfactant before the first inflation at birth improves spatial distribution of ventilation and reduces lung injury in preterm lambs. J Appl Physiol. (2014) 116:251–8. 10.1152/japplphysiol.01142.2013 24356523

[24] Tingay DG Rajapaksa A Zannin E Pereira-Fantini PM Dellaca RL Perkins EJ. Effectiveness of individualized lung recruitment strategies at birth: an experimental study in preterm lambs. Am J Physiol Lung Cell Mol Physiol. (2017) 312:L32–41. 10.1152/ajplung.00416.2016 27881405

[25] Roberts D Dalziel S. Antenatal corticosteroids for accelerating fetal lung maturation for women at risk of preterm birth. Cochrane Database Syst Rev. (2006) 3:CD004454. 10.1002/14651858.CD004454.pub2 16856047

[26] Schneider CA Rasband WS Eliceiri KW. NIH image to imageJ: 25 years of image analysis. Nat Meth. (2012) 9:671–5. 10.1038/nmeth.2089 22930834 PMC5554542

[27] Doyle LW Faber B Callanan C Freezer N Ford GW Davis NM. Bronchopulmonary dysplasia in very low birth weight subjects and lung function in late adolescence. Pediatrics. (2006) 118:108–3. 10.1542/peds.2005-2522 16818555

[28] Reyburn B Martin RJ Prakash YS MacFarlane PM. Mechanisms of injury to the preterm lung and airway: implications for long-term pulmonary outcome. Neonatology. (2012) 101:345–52. 10.1159/000337355 22940624 PMC3567481

[29] Fawke J Lum S Kirkby J Hennessy E Marlow N Rowell V. Lung function and respiratory symptoms at 11 years in children born extremely preterm: the EPICure study. Am J Respir Crit Care Med. (2010) 182:237–45. 10.1164/rccm.200912-1806OC 20378729 PMC2913237

[30] Welsh L Kirkby J Lum S Odendaal D Marlow N Derrick G. The EPICure study: maximal exercise and physical activity in school children born extremely preterm. Thorax. (2010) 65:165–72. 10.1136/thx.2008.107474 19996340

[31] Been JV Lugtenberg MJ Smets E van Schayck CP Kramer BW Mommers M. Preterm birth and childhood wheezing disorders: a systematic review and meta-analysis. PLoS Med. (2014) 11:e1001596. 10.1371/journal.pmed.1001596 24492409 PMC3904844

[32] Ahlfeld SK Conway SJ. Assessment of inhibited alveolar-capillary membrane structural development and function in bronchopulmonary dysplasia. Birth Defects Res A Clin Mol Teratol. (2014) 100:168–79. 10.1002/bdra.23226 24604816 PMC3999962

[33] Thibeault DW Mabry SM Norberg M Truog WE Ekekezie II. Lung microvascular adaptation in infants with chronic lung disease. Biol Neonate. (2004) 85:273–82. 10.1159/000076388 14739556

[34] Hislop A Wigglesworth JS Desai R. Alveolar development in the human fetus and infant. Early Hum Dev. (1986) 13:1–11. 3956418 10.1016/0378-3782(86)90092-7

[35] Kemp MW Saito M Usuda H Watanabe S Sato S Hanita T. The efficacy of antenatal steroid therapy is dependent on the duration of low-concentration fetal exposure: evidence from a sheep model of pregnancy. Am J Obstet Gynecol. (2018) 219:301 e1–16. 10.1016/j.ajog.2018.05.007 29758177

[36] Milesi I Tingay DG Zannin E Bianco F Tagliabue P Mosca F. Intratracheal atomized surfactant provides similar outcomes as bolus surfactant in preterm lambs with respiratory distress syndrome. Pediatr Res. (2016) 80:92–100. 10.1038/pr.2016.39 26954481

[37] Jobe H Hillman N Polglase G Kramer BW Kallapur S Pillow J. Injury and inflammation from resuscitation of the preterm infant. Neonatology. (2008) 94:190–6. 10.1159/000143721 18832854

[38] Burri PH. Fetal and postnatal development of the lung. Annu Rev Physiol. (1984) 46, 617–28. 10.1146/annurev.ph.46.030184.003153 6370120

[39] Vlahovic G Russell ML Mercer RR Crapo JD. Cellular and connective tissue changes in alveolar septal walls in emphysema. Am J Respir Crit Care Med. (1999) 160:2086–92. 10.1164/ajrccm.160.6.9706031 10588633

[40] Egberts J Gorree GC Boonman AA. Lack of change in the composition of fetal lamb lung surfactant during gestation. Biochim Biophys Acta. (1986) 878:146–51. 3756189 10.1016/0005-2760(86)90140-2

[41] Kothe TB Royse E Kemp MW Usuda H Saito M Musk GC Epidermal growth factor receptor inhibition with gefitinib does not alter lung responses to mechanical ventilation in fetal, preterm lambs. PLoS ONE. (2018) 13:e0200713 10.1371/journal.pone.0200713 30005089 PMC6044532

[42] Joss-Moore A Hagen-Lillevik SJ Yost C Jewell J Wilkinson RD Bowen S Alveolar formation is dysregulated by restricted nutrition but not excess sedation in preterm lambs managed by noninvasive support. Pediatr Res. (2016) 80:719–28. 10.1038/pr.2016.143 27429203 PMC5683895

[43] Deptula N Royse E Kemp MW Miura Y Kallapur SG Jobe AH. Brief mechanical ventilation causes differential epithelial repair along the airways of fetal, preterm lambs. Am J Physiol Lung Cell Mol Physiol. (2016) 311:L412–20. 10.1152/ajplung.00181.2016 27343193 PMC5142451

[44] Polglase R Barton SK Melville JM Zahra V Wallace MJ Siew ML. Prophylactic erythropoietin exacerbates ventilation-induced lung inflammation and injury in preterm lambs. J Physiol. (2014) 592:1993–2002. 10.1113/jphysiol.2013.270348 24591575 PMC4230774

[45] Hillman NH Moss TJ Nitsos I Jobe AH. Moderate tidal volumes and oxygen exposure during initiation of ventilation in preterm fetal sheep. Pediatr Res. (2012) 72:593–9. 10.1038/pr.2012.135 23037872 PMC4073615

[46] Hillman NH Nitsos I Berry C Pillow JJ Kallapur SG Jobe AH. Positive end-expiratory pressure and surfactant decrease lung injury during initiation of ventilation in fetal sheep. Am J Physiol Lung Cell Mol Physiol. (2011) 301:L712–20. 10.1152/ajplung.00157.2011 21856815 PMC3290453

[47] Mariani L Jonusas SF Maure C Esteban M Pardo A Rapetti B. Open lung strategy in a lamb model of respiratory distress syndrome. Am J Perinatol. (2011) 28:585–92. 10.1055/s-0031-1275385 21425032

[48] Ball K Hillman NH Kallapur SG Polglase GR Jobe AH Pillow JJ. Body temperature effects on lung injury in ventilated preterm lambs. Resuscitation. (2010) 81:749–54. 10.1016/j.resuscitation.2009.12.007 20299144 PMC2871967

[49] Sato A Whitsett JA Scheule RK Ikegami M. Surfactant protein-d inhibits lung inflammation caused by ventilation in premature newborn lambs. Am J Respir Crit Care Med. (2010) 181:1098–5. 10.1164/rccm.200912-1818OC 20133924 PMC2874451

[50] Miller TL Shashikant BN Pilon AL Pierce RA Shaffer TH Wolfson MR. Effects of recombinant clara cell secretory protein (rhCC10) on inflammatory-related matrix metalloproteinase activity in a preterm lamb model of neonatal respiratory distress. Pediatr Crit Care Med. (2007) 8:40–6. 10.1097/01.PCC.0000253022.10607.61 17149150

[51] Crossley KJ Morley CJ Allison BJ Polglase GR Dargaville PA Harding R. Blood gases and pulmonary blood flow during resuscitation of very preterm lambs treated with antenatal betamethasone and/or curosurf. effect of positive end-expiratory pressure. Pediatr Res. (2007) 62:37–42. 10.1203/PDR.0b013e31806790ed 17515834

[52] Musk GC Polglase GR Bunnell JB Nitsos I Tingay D Pillow JJ. A comparison of high-frequency jet ventilation and synchronised intermittent mandatory ventilation in preterm lambs. Pediatr Pulmonol. (2015) 50:1286–93. 10.1002/ppul.23187 25823397

[53] Polglase GR Tingay DG Bhatia R Berry CA Kopotic RJ Kopotic CP. Pressure-versus volume-limited sustained inflations at resuscitation of premature newborn lambs. BMC Pediatr. (2014) 14:43. 10.1186/1471-2431-14-43 24529320 PMC3937019

[54] Hillman H Gisslen T Polglase GR Kallapur SG Jobe AH Ventilation-induced increases in EGFR ligand mRNA are not altered by intra-amniotic LPS or ureaplasma in preterm lambs. PLoS ONE. (2014) 9:e96087 10.1371/journal.pone.0096087 24788984 PMC4005755

[55] Hillman H Kemp MW Miura Y Kallapur SG Jobe AH Sustained inflation at birth did not alter lung injury from mechanical ventilation in surfactant-treated fetal lambs. PLoS ONE. (2014) 9:e113473 10.1371/journal.pone.0113473 25419969 PMC4242618

[56] Jobe H. What is BPD in 2012 and what will BPD become? Early Hum Dev. (2012) 88(Suppl 2):S27–8. 10.1016/S0378-3782(12)70009-9 22633507

