# Supplementary material for: Gestational Age Influences the Early Microarchitectural Changes in Response to Mechanical Ventilation in the Preterm Lamb Lung
Source: Front Pediatr. 2019 Aug 21;7:325. doi: 10.3389/fped.2019.00325 (PMC6712425; doi:10.3389/fped.2019.00325)
Supplement: Supplementary file 1 [file Data_Sheet_1.pdf]

Supplementary Material

1 Supplementary Data

2 METHODS

In automated assessments the following macro codes were installed as plugins in ImageJ;

% lung tissue;

```
run("Set Scale...", "distance=3 known=1 pixel=1 unit=um global");

run("8-bit");

setAutoThreshold("Default");

//run("Threshold...");

run("Measure");
```

Data used for qualitative alveolar assessments including number of alveolar, total alveolar area, and average alveolar area was generated using the following macro script;

```
run("Set Scale...", "distance=3 known=1 pixel=1 unit=um global");

run("Subtract Background...", "rolling=50 light");

run("Sharpen");

run("Enhance Contrast...", "saturated=0.4");
```

```
run("Find Edges");
run("Gaussian Blur...", "sigma=2");
setOption("BlackBackground", false);
run("Make Binary");
run("Close-");
run("Invert");
run("Analyze Particles...", "size=235-Infinity show=[Overlay Outlines] display");
```

3 Supplementary Figures and Tables

Supplementary Table 1: Reporting of the 95% **c**onfidence interval of mean from study 1.

	**124d**		**128d**	
	**Unventilated**	**Ventilated**	**Unventilated**	**Ventilated**
Actual confidence level	93.8%	96.1%	93.8%	98.7%
% lung tissue	1.1 – 1.6	1.3 – 2.9	1.1 – 1.4	1.6 – 2.0
Alveolar septal width	14.0 – 23.9	6.9 – 8.8	14 – 26.5	6.7 – 9.3
Alveolar units	531 - 642	446 - 529	480 - 737	398 – 498
Average alveolar area	914 - 1668	1410 - 1717	978 - 1225	1585 – 2118
Variance in alveolar area	1.1 – 1.6	1.3 – 2.9	1.1 – 1.4	1.6 – 2.0
Total alveolar area	563 - 936	664 - 743	571 - 756	737 – 817
Detached epithelial cells	2 - 11	16 - 42	1 - 6	1 – 19

Supplementary Table 2: Reporting of the 95% **c**onfidence interval of mean from study 2.

	119d	124d	128d	133d	140d
Actual confidence level	98.4%	96.1%	96.5%	97.9%	97.9%
% lung tissue	25.7 – 30.1	26.7 – 30.0	23.6 – 26.2	23.3 – 26.4	21.5 – 24.9
Alveolar septal width	6.5 – 12.4	6.9 – 8.8	6.9 – 9.3	4.5 – 6.5	4.2 – 5.9
Alveolar units	362 - 483	446 - 529	404 - 497	320 - 439	403 – 467
Average alveolar area	612 - 715	664 - 743	722 - 810	736 - 820	774 – 812
Variance in alveolar area	1.4 – 1.8	1.3 – 2.9	1.7 – 2.0	1.8 – 2.3	1.6 – 2.0
Total alveolar area	1377 - 1838	1410 - 1717	1552 - 2073	1842 - 2666	1733 – 2086
Detached epithelial cells	11.4 – 35.0	16.0 – 41.2	2.4 – 21.0	3.2 – 5.5	1.1 – 21.4

4 **Supplementary Table 3**: Respiratory characteristics at conclusion of Study 3.

		119d	124d	128d	133d	140d	P value (ANOVA)
**Ventilator parameters**	**PIP (cm H_2O)**	28 (25, 35)	30 (22, 35)	24 (22, 32)	21 (18, 26)*†	21 (17, 35)	0.0004
	**V_T (ml/kg)**	7.5 (6.7, 11.7)	6.7 (5.9, 7.9)	6.6 (6.0, 7.2)	6.1 (4.6, 7.2)*	5.5 (4.5, 8.1)**	0.0013
**Gas exchange**	**FiO_2**	0.50 (0.30, 0.75)	0.38 (0.21, 1.00)	0.22 (0.21, 0.45)	0.21 (0.21, 0.80)*†	0.21 (0.21, 0.21)**††	<0.0001

	**$AaDO_2$**	245 (120, 413)	168 (58, 638)	61 (27, 208)	37 (22, 339)*†	16 (6, 29)**††‡‡	<0.0001
	**$PaCO_2$**	56.6 (45.9, 58.5)	49.6 (42.4, 60.6)	48.4 (42.8, 60.6)	51.8 (39.2, 56.4)	43.7 (33.6, 50.5)**	0.0042
**Lung mechanics**	**Static compliance (ml/kg/cm H_2O)**	1.06 (0.56, 1.25)	1.00 (0.00, 1,71)	1.13 (0.66, 1.71)	1.23 (1.02, 1.40)	1.31 (0.91, 1.59)	0.0971
**Markers of lung injury**	**Lung fluid protein concentration (mg/µl)**	427.7 (328.1, 445.9)	493.1 (292.2, 863.8)	381.6 (225.2, 1074.0)	210.5 (118, 499.5)†	245.6 (109.9, 534.5)††	0.0030
	**Relative EGR1 gene expression (ΔΔCt)**	1.70 (0.64, 7.30)	1.59 (0.32, 4.16)	1.24 (0.32, 5.28)	0.59 (0.35, 2.93)	1.43 (0.32, 2.45)	0.0902
	**Relative CYR61 gene expression (ΔΔCt)**	0.71 (0.47, 1.17)	0.75 (0.29, 1.08)	0.69 (0.28, 2.00)	0.95 (0.51, 2.59)	1.01 (0.42, 2,44)	0.1943
	**Relative CTGF gene expression (ΔΔCt)**	0.46 (0.28, 0.84)	0.74 (0.26, 2.41)	0.54 (0.33, 1.26)	0.98 (0.66, 2.32)	1.25 (0.36, 1.41)	0.0189
	**Relative IL1B gene expression (ΔΔCt)**	2.58 (1.12, 5.78)	1.83 (0.44, 7.12)	2.90 (0.82, 10.76)	1.23 (0.48, 7.54)	1.30 (0.18, 6.54)	0.1092
	**Relative IL6 gene expression (ΔΔCt)**	4.93 (3.59, 23.43)	4.19 (1.89, 5.52)	4.83 (2.02, 17.84)	1.37 (0.58, 7.99)*‡	1.14 (0.19, 1.98)**††‡‡	<0.0001
	**Relative IL8 gene expression (ΔΔCt)**	34.98 (4.6, 44.89)	9.70 (0.33, 51.65)	24.01 (0.78, 202.30)	23.26 (0.36, 379.5)	5.55 (0.54, 48.2)	0.1964

Abbreviations: Peak inspiratory pressure (PIP), tidal ventilation (V_T), Fraction of inspired oxygen (FiO_2), alveolar-arterial oxygen difference ($AaDO_2$), partial pressure of carbon dioxide ($PaCO_2$), Early growth response 1 (EGR1), Cysteine-rich angiogenic inducer 61 (CYR61), Connective tissue growth factor (CTGF), Interleukin-1beta (IL1B), Interleukin-6 (IL6), Interleukin-8 (IL8). Data represented as median (IQR). $P < 0.05$ for comparison of: *119d vs 133d, **119d vs 140d, †124d vs 133d, ††124d vs 140d, ‡128d vs 133d, ‡‡128d vs 140d.
